# Supplementary material for: Three-Dimensional Stable Alginate-Nanocellulose Gels for Biomedical Applications: Towards Tunable Mechanical Properties and Cell Growing
Source: Nanomaterials (Basel). 2019 Jan 8;9(1):78. doi: 10.3390/nano9010078 (PMC6359031; doi:10.3390/nano9010078)
Supplement: Supplementary file 1 [file nanomaterials-09-00078-s001.pdf]

Three-dimensional stable alginate-nanocellulose gels for biomedical applications: towards tunable mechanical properties and cell growing

Priscila Siqueira [a], Éder Siqueira [b], Ana Elza de Lima [b], Gilberto Siqueira [c], Ana Délia Pinzón Garcia [b], Ana Paula Lopes [b], Maria Esperanza Cortés Segura [d], Augusta Isaac [e], Fabiano Vargas Pereira [b], Vagner Roberto Botaro [f] **

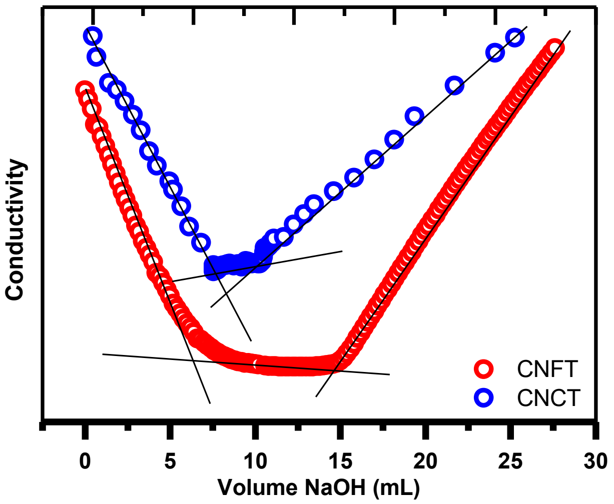

Figure S1: Conductometric titration curves for TEMPO-oxidized cellulose nanofibers (CNFT) and TEMPO-oxidized cellulose nanocrystals (CNCT)

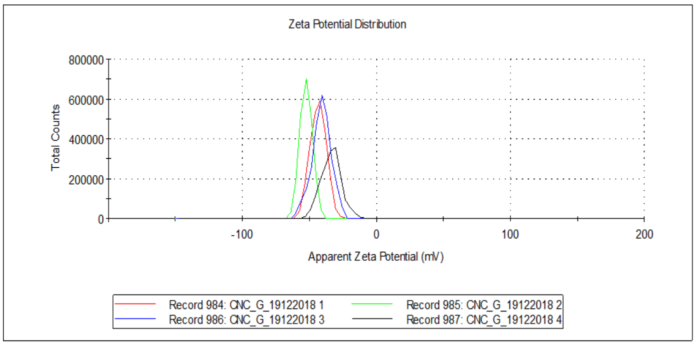

CNC

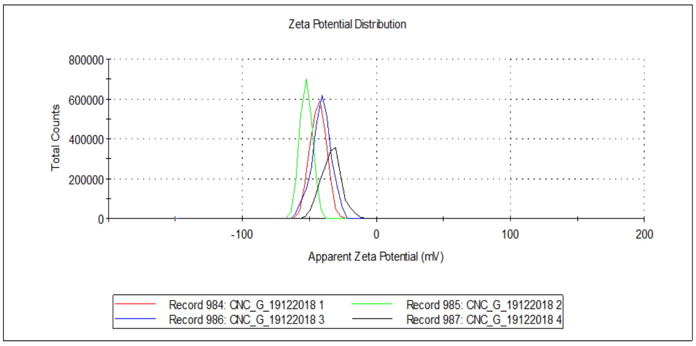

CNCT

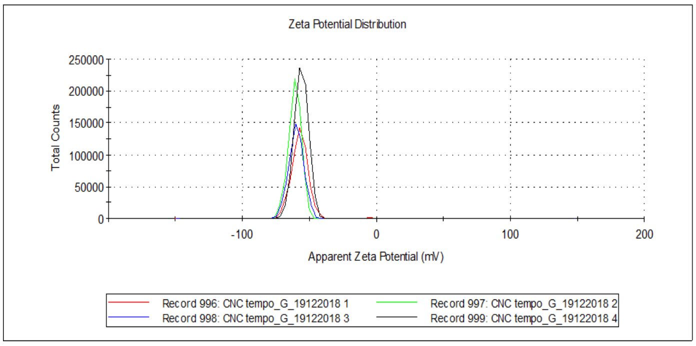

CNF

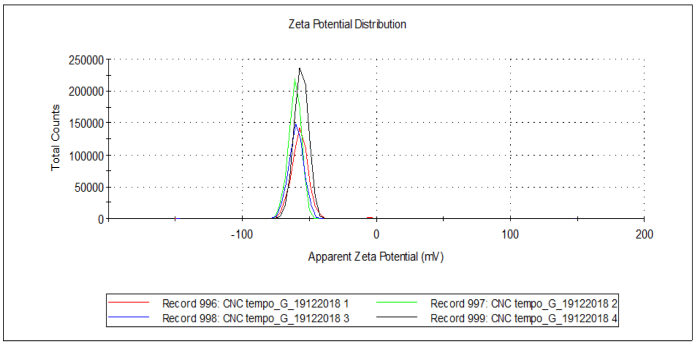

CNFT

Figure S2: Zeta potencial (ζ) measurements obtained by electrophoretic mobility for CNC, CNCT, CNF and CNFT.

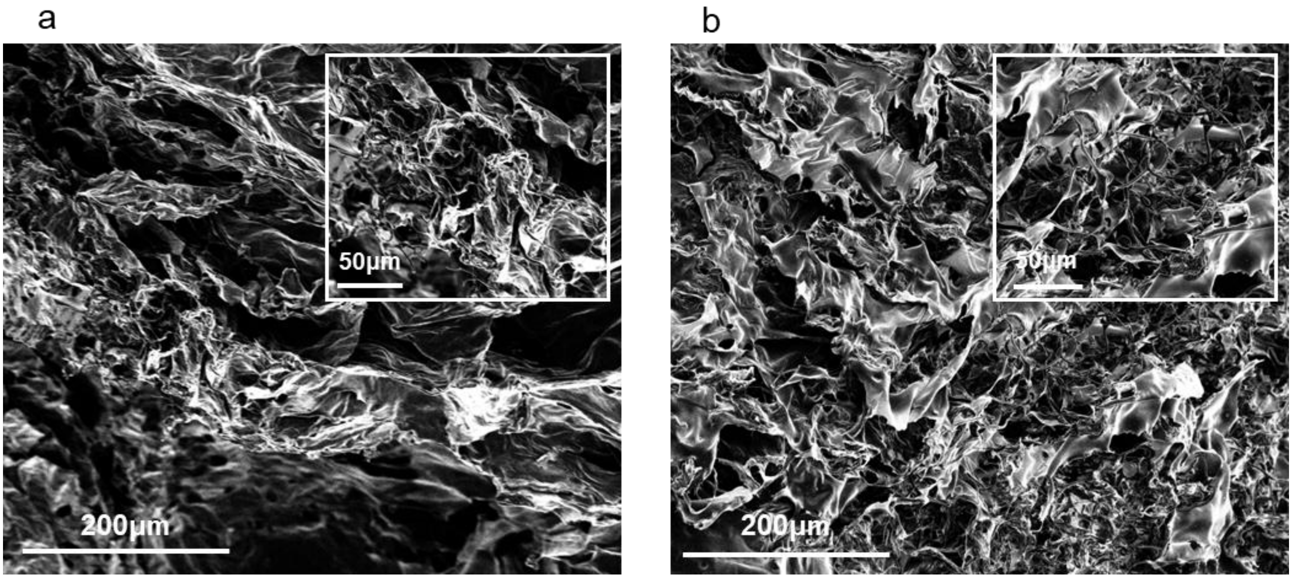

Figure S3: SEM images of the cross-section for crosslinked gels (a) alginate/CNC 50 wt% and 500 x magnification; (b) alginate/CNF 50 wt% 500 x magnification. The inset represents a magnification of 1500 x.

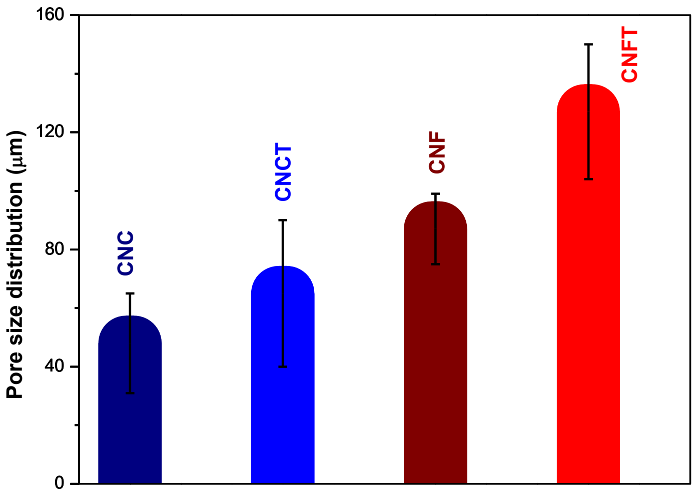

Figure S4: Pore sizes distribution and standard mean values obtained by SEM micrographs for CNC, CNCT, CNF and CNFT.

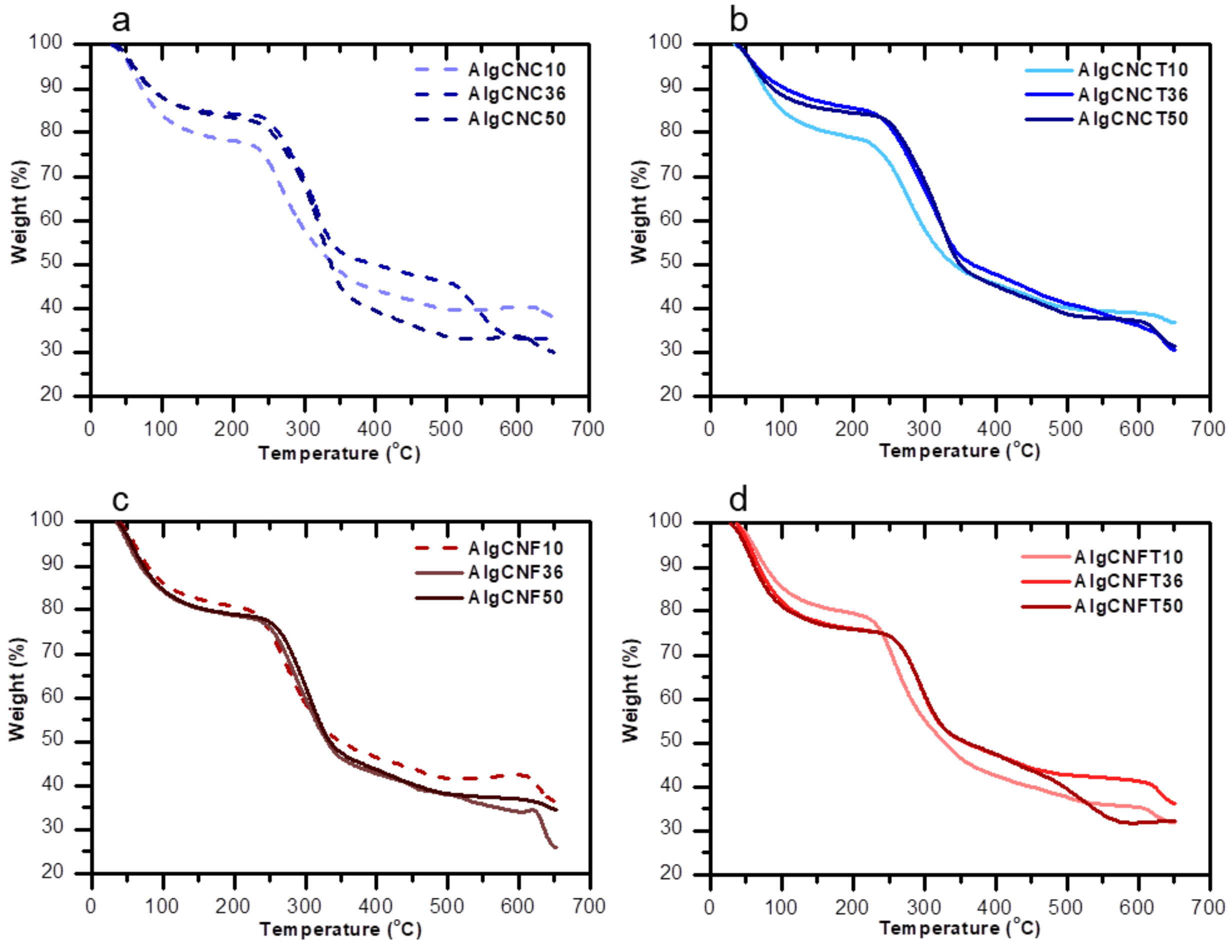

Figure S5: Influence of the nanocellulose concentration on the thermal stability of aginate-gels: (a) CNC (10, 36 and 50 wt%); (b) CNCT (10, 36 and 50 wt%); (c) CNF (10, 36 and 50 wt%); (d) CNFT (10, 36 and 50 wt%).
